# MDM2 knockdown mediated by a triazine-modified dendrimer in the treatment of non-small cell lung cancer

**DOI:** 10.18632/oncotarget.9768

**Published:** 2016-06-01

**Authors:** Quan Huang, Lei Li, Lin Li, Hui Chen, Yongyan Dang, Jishen Zhang, Naimin Shao, Hong Chang, Zhengjie Zhou, Chongyi Liu, Bingwei He, Haifeng Wei, Jianru Xiao

**Affiliations:** ^1^ Department of Orthopedic Oncology, Changzheng Hospital, The Second Military Medical University, Shanghai, PR China; ^2^ Shanghai Key Laboratory of Regulatory Biology, School of Life Sciences, East China Normal University, Shanghai, PR China

**Keywords:** dendrimer, MDM2, gene delivery, siRNA, non-small cell lung cancer

## Abstract

Non-small cell lung cancer (NSCLC) is the most common type of lung cancer and the five-year survival rate is lower in advanced NSCLC patients. Chemotherapy is a widely used strategy in NSCLC treatment, but is usually limited by poor therapeutic efficacy and adverse effects. Therefore, a new therapeutic regimen is needed for NSCLC treatment. Gene therapy is a new strategy in the treatment of NSCLC. However, the lack of efficient and low toxic vectors remains the major obstacle. Here, we developed a biocompatible dendrimer as a non-viral vector for the delivery of mouse double minute2 (MDM2) siRNA *in vitro* and *in vivo* to treat NSCLC. The triazine-modified dendrimer efficiently stimulates the down-regulation of MDM2 gene in NSCLC PC9 cells, which induces significant cell apoptosis through the activation of apoptosis markers such as caspase-8 and poly(ADP-ribose) polymerase (PARP) cleavage. Furthermore, the dendrimer/MDM2 siRNA polyplexes showed excellent activity in the inhibition of tumor growth in a PC9 xenograft tumor model. These results suggested that inhibition the expression of MDM2 might be a potential target in NSCLC treatment.

## INTRODUCTION

Lung cancer is the most common cancer globally, with more than 1.8 million people diagnosed with the condition each year [1, 2]. It is the leading cause of cancer-related death in man [3]. There are two different lung cancer subtypes including small-cell lung cancer (SCLC) and non-small-cell lung cancer (NSCLC) according to their physiological phenotypes. NSCLC is ranked the majority in lung cancer patients, which accounts for over 85% of clinical lung cancer cases [4, 5]. It includes squamous-cell carcinoma, adenocarcinoma and large-cell carcinoma. The five-year survival rate is less than 5% in advanced NSCLC patients. Chemotherapy after surgery, known as adjuvant chemotherapy, is a widely used strategy in NSCLC treatment [6, 7]. However, for patients with multidrug resistant NSCLC, the adjuvant chemotherapy is less effective [8]. In addition, chemotherapy is always associated with serious side-effects, which affect the life quality of patients. Therefore, a new therapeutic regimen is needed for NSCLC treatment.

Small interfering RNA (siRNA)-based gene therapy has offered a new avenue in the clinical treatment of NSCLC [9–11]. A key challenge to clinical siRNA therapy is the lack of biocompatible and effective gene vectors [12, 13]. Dendrimers were considered as a class of non-viral vectors for siRNA delivery due to limited immunogenicity, well-defined architecture, and feasibility to surface modification [14–17]. These polymers possess a high density of surface cationic charges for siRNA binding and a large number of tertiary amine groups for pH buffering, which is beneficial for intracellular endosomal escape [18]. For example, high generation (generation 7, G7) polyamidoamine (PAMAM) dendrimers with a triethanolamine core efficiently induced endogenous gene silencing [19, 20]. However, intact dendrimers are usually criticized by moderate siRNA delivery efficacy and non-negligible toxicity. As a result, the surfaces of dendrimers are usually engineered with various ligands to improve their performances in siRNA delivery [21]. Recently, we developed a4,6-diamino-1,3,5-triazine (DAT) modified PAMAM dendrimer that fulfills the two major requirements in DNA delivery—high transfection efficacy and minimal cytotoxicity [22]. This polymer efficiently transfects plasmid encoding tumor necrosis factor (TNF) increased apoptosis inducing ligand (TRAIL) in tumors and inhibits tumor growth in osteosarcoma-bearing mice [23]. However, the efficacy of DAT-modified dendrimer in siRNA delivery is unknown.

Mouse double minute2 (MDM2) functions as an oncogene in tumorigenesis [24]. Gene amplification and protein overexpression of MDM2 are found in a number of human tumors, including NSCLC [25]. As an oncogene, MDM2 protein decreases apoptosis by inhibiting the tumor suppressor gene p53 [26]. In addition, MDM2 is involved in p53-independent carcinogenesis via downregulation of pRb [27]. MDM2 was identified as an Rb-binding protein. Rb is a potent tumor suppressor that is mutated in different kinds of cancers. MDM2 inhibits the ability of Rb to inhibit E2F1 function, thus inhibiting arrest of the cell cycle in G1 [24, 28, 29]. The binding of MDM2 to p53 promotes its degradation via ubiquitin and inhibits its transcriptional activation [30]. In addition, MDM2 gene amplification is considered as an factor of adverse prognosis in NSCLC [31]. Therefore, MDM2 can be considered as a potential target for NSCLC therapy.

In this study, we investigated the potential use of DAT-modified dendrimer in the delivery of siRNA specifically inhibiting MDM2 (siMDM2) in the treatment of NSCLC. Downregulation of MDM2 by the developed polyplexes induced significant apoptosis of NSCLC PC9 cells *in vitro*, and inhibited the tumor growth in a PC9 xenograft tumor model. The aim of this study is to provide a non-viral gene therapy approach for NSCLC therapy.

## RESULTS

### Characterization of dendrimer/siRNA-MDM2 polyplexes

**Figure 1:**
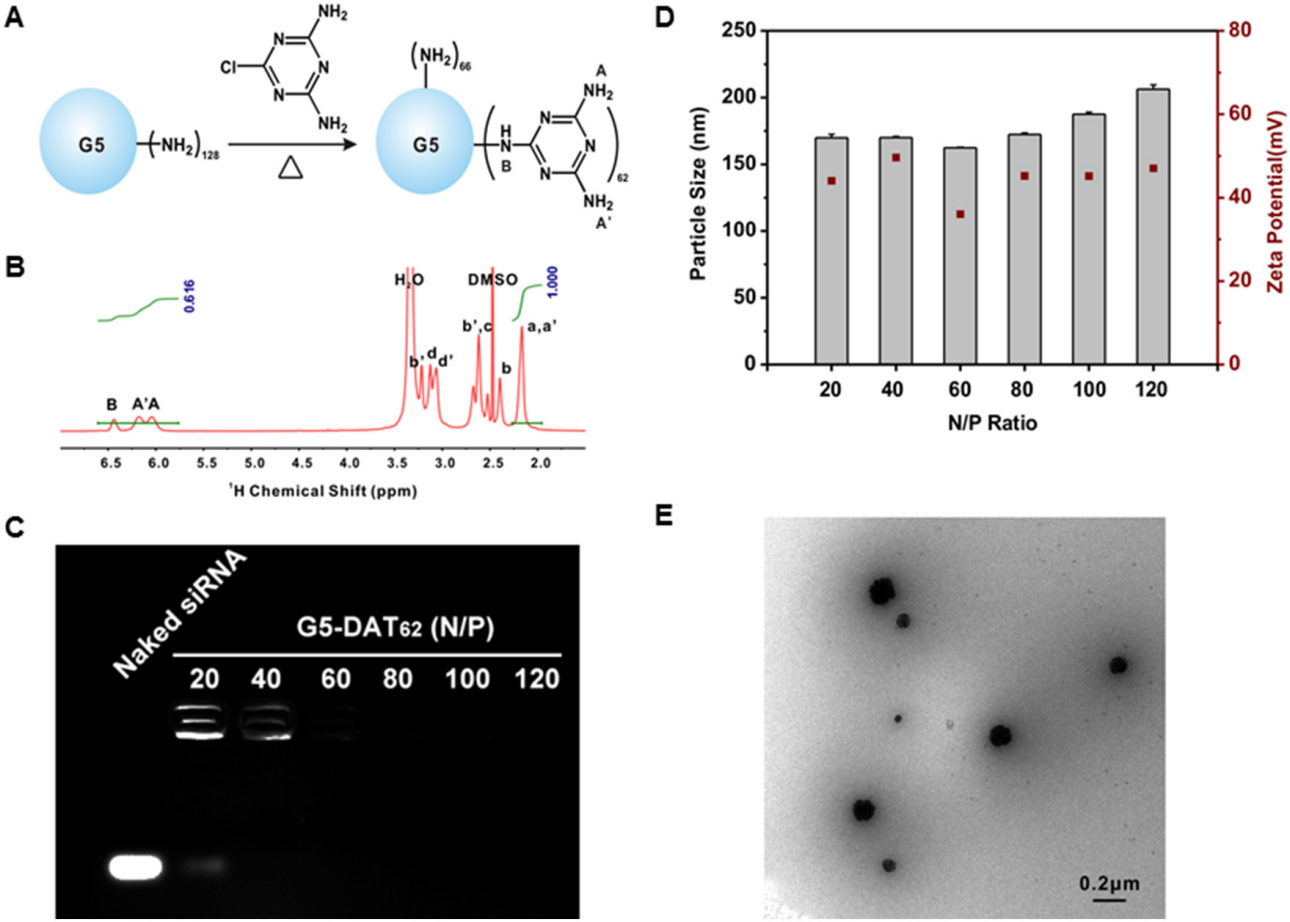
Synthesis and characterization of G5-DAT_62_ and its complexes with siRNA **A.** Synthesis of G5-DAT_62_ by a facile reaction. **B.** ^1^H NMR spectrum of the synthesized G5-DAT_62_ in DMSO-D6. The number of DAT molecules conjugated on the surface of each G5 PAMAM dendrimer was calculated according to the intensities of dendrimer protons (H_a,a'_) and DAT protons (H_A,A',B_). Protons a-d are the interior methylene protons and protons a'-d' are the methylene protons on the outmost layer of a G5 PAMAM dendrimer, respectively. **C.** siRNA binding capacity of G5-DAT_62_ evaluated by agarose gel electrophoresis. The polyplexes were prepared at N/P ratios of 20, 40, 60, 80, 100 and 120, respectively. **D.** Characterization of G5-DAT_62_/siRNA polyplexes using dynamic light scattering and zeta potential at different N/P ratios. **E.** TEM image of G5-DAT_62_/siRNA polyplexes prepared at an N/P ratio of 100.

The DAT-modified G5 PAMAM dendrimer is synthesized by a facile method as shown in Figure 1A. According to the ^1^H NMR spectrum, an average number of 62 DAT molecules were conjugated on the surface of each G5 PAMAM dendrimer (Figure 1B). The yielding material is termed G5-DAT_62_. The siMDM2 binding capacity of G5-DAT_62_ was evaluated by an agarose gel retardation assay (Figure 1C). At an N/P ratio above 20, G5-DAT_62_ is able to completely retard the mobility of siMDM2. The formed G5-DAT_62_/siMDM2 polyplexes were further characterized by DLS (Figure 1D) and TEM (Figure 1E). It is observed that G5-DAT_62_ was able to form positively charged nanoparticles below 200 nm with siMDM2 (N/P ratio above 20). TEM image further confirmed nanoscale size of G5-DAT_62_/siMDM2 polyplexes at an N/P ratio of 100.

### MDM2 knockdown in PC9 cells promotes cell apoptosis

**Figure 2:**
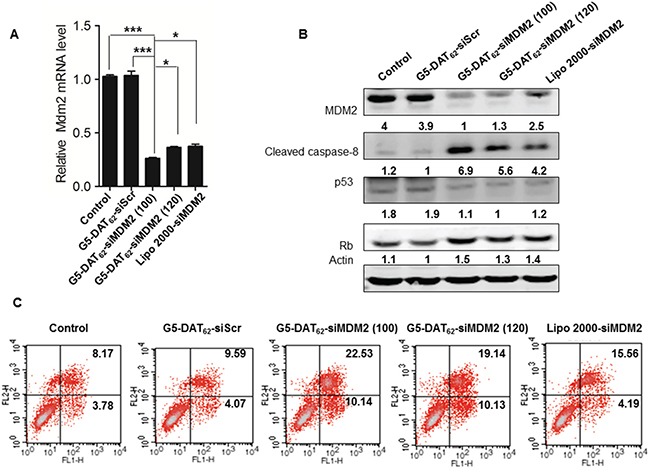
MDM2 knockdown and apoptosis induction in NSCLC PC9 cells **A.** MDM2 mRNA expressions in PC9 cells treated with G5-DAT_62_/siScr, G5-DAT_62_/siMDM2 (N/P = 100 and 120), and Lipo 2000/siMDM2 determined by quantitative real time PCR. * p < 0.05 and ***p < 0.001. **B.** Representative cleaved caspase-8, p53 and Rb protein expressions in the transfected PC9 cells determined by western blot analysis. **C.** Apoptosis analysis of the transfected PC9 cells by an Annexin V-FITC/PI double staining assay.

To study whether MDM2 knockdown could induce a sufficient apoptotic response for NSCLC, we transfected the NSCLC PC9 cells with G5-DAT_62_/siMDM2 polyplexes. As shown in Figure 2A, the G5-DAT_62_/siMDM2 polyplexes prepared at N/P ratios of 100 and 120 significantly decreased the expression of MDM2 mRNA in PC9-luc cells. At an optimal N/P ratio of 100, more than 70% of MDM2 expression was silenced, which is even more efficient than the commercial transfection reagent Lipo 2000. In comparison, the control polyplexes (G5-DAT_62_/siScr) failed to knockdown the MDM2 mRNA. Western blot analysis in Figure 2B also revealed that the expression of MDM2 protein in the PC9-luc cells transfected by G5-DAT_62_/siMDM2 and Lipo 2000/siMDM2 is significantly inhibited. To confirm the apoptotic responses in PC9-luc cells caused by MDM2 suppression, we analyzed the transfected cells by an Annexin V-FITC/PI apoptosis detection kit. As shown in Figure 2C, transfection of PC9-luc cells with G5-DAT_62_/siMDM2 polyplexes at an N/P ratio of 100 caused a higher percent of apoptotic cells than those with G5-DAT_62_/siScr and Lipo 2000/siMDM2.

As an oncogene, MDM2 protein decreases apoptosis by inhibiting the tumor suppressor gene p53 [26]. The binding of MDM2 to p53 promotes its degradation via ubiquitin and inhibits its transcriptional activation [30]. Alternatively, MDM2 is involved in p53-independent carcinogenesis via downregulation of pRb [27]. MDM2 was identified as an Rb-binding protein. Rb is a potent tumor suppressor that is mutated in different kinds of cancers. MDM2 inhibits the ability of Rb to inhibit E2F1 function, thus inhibiting arrest of the cell cycle in G1 [28, 29]. Considering that p53 is mutated in PC9 cells, we assumed that MDM2 repression in PC9 cells induces apoptosis through a p53-independent pathway according to regulating the expression of Rb. To confirm this hypothesis, we analyzed the expressions of p53 and Rb proteins in the transfected PC9 cells by western blot. As shown in Figure 2B, silencing MDM2 in PC9 cells had a slight effect on p53, but increased the Rb level. Besides, the well-known caspase-8 apoptosis pathway is activated after MDM2 knockdown. These results suggest that MDM2 can be considered as a potential target for NSCLC therapy and G5-DAT_62_ can be used as efficient vector for the delivery of siMDM2 into NSCLC PC9 cells.

### Transfection mechanism and biocompatibility of G5-DAT_62_

**Figure 3:**
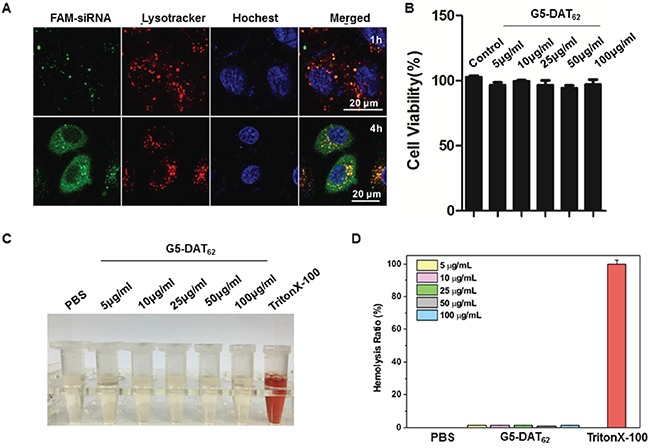
Transfection mechanism and biocompatibility of G5-DAT_62_ **A.** Confocal images of PC9 cells incubated with G5-DAT_62_/FAM-siRNA polyplexes (green) at an N/P ratio of 100 for 1 h and 4 h, respectively. The endosomes were stained with LysoTracker Red (red) and the nuclei were stained with Hoechst 33342 (blue). **B.** Viability of PC9 cells treated with G5-DAT_62_ at different concentrations. The cells were incubated with the material for 24 h. **C.** Photographs of supernatant of the red blood cells solution treated with G5-DAT_62_ at different concentrations. PBS and Triton X-100 (0.5%) were used as negative and positive controls, respectively. **D.** Hemolysis ratio statistics of G5-DAT_62_ at concentrations ranging from 5 to 100 μg/mL.

Efficacy of a polymeric vector in siRNA delivery depends on multiple parameters, including polyplex formation, cellular uptake, endosomal escape, and intracellular siRNA release. As shown in Figure 1D and Figure 1E, G5-DAT_62_ is able to stably bind siRNA and condense them into polyplexes around 200 nm, which is beneficial for efficient gene delivery. In addition, DAT-modified G5 dendrimer can efficiently deliver plasmid DNA or siRNA into different cell lines [22]. We further investigated the endosomal escape behaviors of G5-DAT_62_/siMDM2 polyplexes after cellular uptake. As shown in Figure 3A, the FAM-labeled G5-DAT_62_/siRNA polyplexes (green) are entrapped within the acidic vesicles stained by Lysotracker (red) at 1 h, but most of the nanoparticles successfully escaped from the acidic vesicles after 4 h incubation. This is probably due to the fact that G5 PAMAM dendrimer has a large number of tertiary amine groups on the polymer scaffold, which facilitates the endosomal escape of G5-DAT_62_/siRNA polyplexes through a well-known proton-sponge mechanism.

G5-DAT_62_ causes minimal toxicity to PC9 cells at concentrations up to 100 μg/mL (Figure 3B). More than 90% of the PC9 cells survive after incubation with polymer. The low toxicity of G5-DAT_62_ on the cells can be explained by reduced charge density on G5 PAMAM dendrimer after DAT modification. In addition, G5-DAT_62_ shows minimal hemolytic toxicity (<1%) on the red blood cells (Figure 3C and Figure 3D). These results suggest that G5-DAT_62_ can efficiently deliver siRNA into PC9 cells with minimal cytotoxicity and hemolytic toxicity.

### MDM2 knockdown inhibits tumor growth *in vivo*

**Figure 4:**
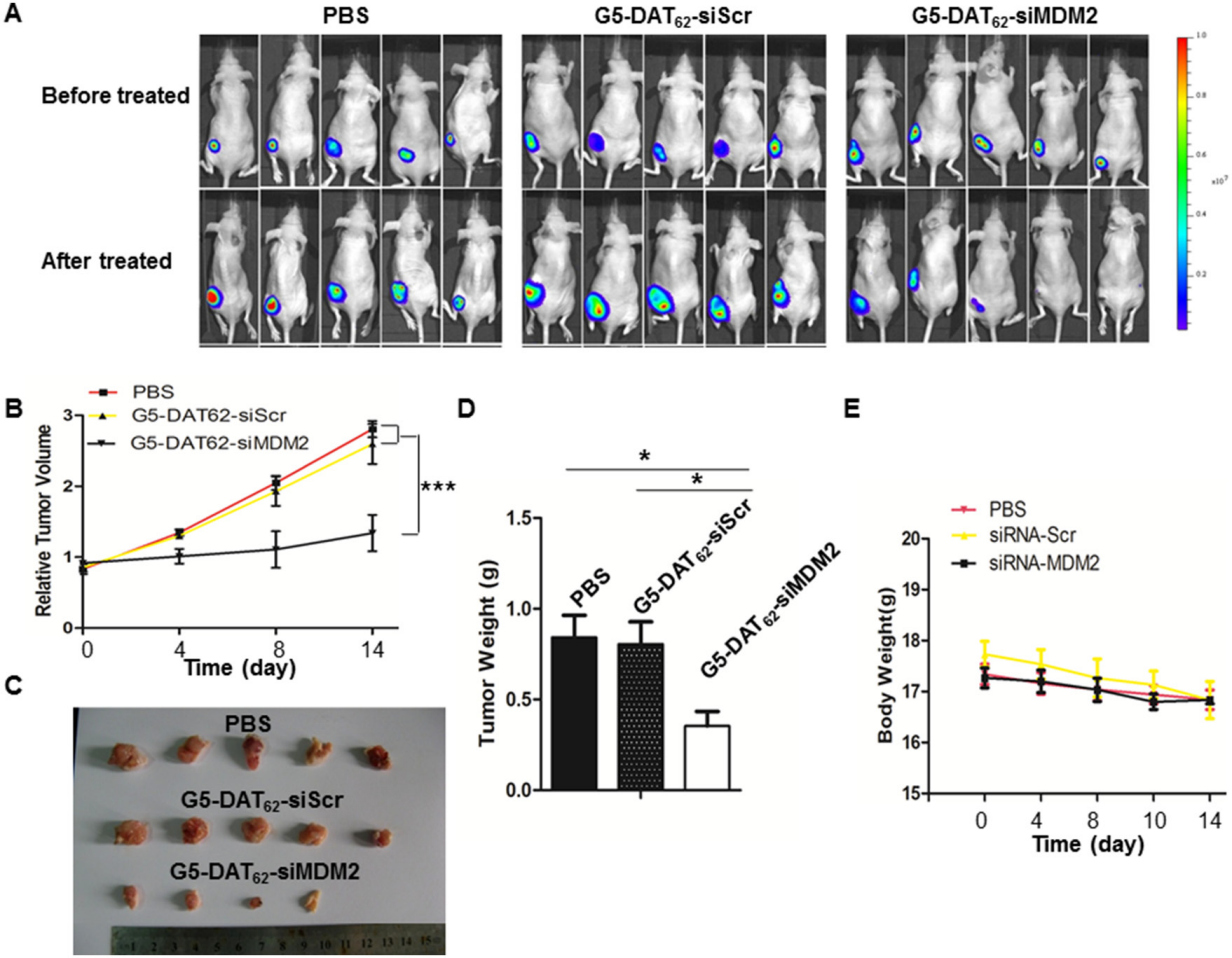
Tumor inhibition by MDM2 knockdown *in vivo* **A.** *in vivo* luminescence images of mice bearing PC9-luc tumors before and after treatment with PBS, G5-DAT_62_/siScr or G5-DAT_62_/siMDM2. **B.** The evolution of tumor volumes during the therapeutic period. **C.** The photograph of the excised tumors after treatment. **D.** The weight of the excised tumors after treatment. **E.** Body weights of the mice during the treatment. *p< 0.05 by student's t-test (n=5).

We further investigated the anti-tumor activity of G5-DAT_62_/siMDM2 polyplexes in a PC9-luc xenograft tumor model. The treatments were initiated when the PC9-luc tumors reached a volume around 200 mm^3^. Figure 4A shows luminescence images of the mice before and after the treatment. The significantly increased luminescence intensities of animals in the PBS group and the G5-DAT_62_/siScr group indicate rapid growth of PC9-luc tumors during the treatment, suggesting that the control treatments have negligible suppression on the tumors. In comparison, treatment of G5-DAT_62_/siMDM2 polyplexes markedly inhibited tumor growth in the model. The tumor volume, tumor weight and the final excised tumor images in Figure 4B-4D further confirm that the tumors from mice treated with G5-DAT_62_/siMDM2 polyplexes were significantly reduced compared to those in the control groups. In addition, G5-DAT_62_/siMDM2 treatment induces minimal change in body weight compared to the PBS and G5-DAT_62_/siScr groups (Figure 4E), suggesting low systemic toxicity of G5-DAT_62_ during the treatment. These results indicate that G5-DAT_62_ has a promising potential in the delivery of siMDM2 to treat NSCLC.

### MDM2 knockdown inhibits tumor growth via promoting cell apoptosis

**Figure 5:**
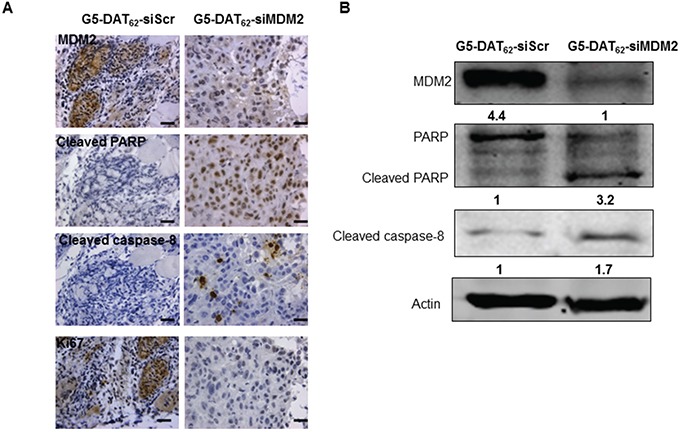
MDM2 knockdown inhibits PC9 tumor growth via promoting apoptosis The levels of MDM2, cleaved PARP cleaved caspase-8 and Ki67 in the tumors after treatment were evaluated by IHC **A.** and western blot **B.** Scar bar in (A) is 25 μm.

To study the mechanism underlying MDM2 knockdown induced tumor suppression, we examined the expressions of MDM2 and cell apoptosis markers in the excised tumors after treatment. Caspase-8 is a member of the cysteine proteases, which are implicated in apoptosis. Active caspase-8 can initiate apoptosis directly by cleaving and thereby activating executioner caspase-3, caspase-6 and caspase-7 to induce efficient cell death [32]. Poly (ADP-ribose) polymerase (PARP) cleavage is often associated with apoptosis and has been served as a hallmark of apoptosis and caspase activation [33]. IHC analysis in Figure 4A showed obvious MDM2 knockdown in PC9-luc xenograft tumor treated with G5-DAT_62_/siMDM2 polyplexes in comparison with the G5-DAT_62_/siScr control. As expected, marked PARP and cleaved caspase-8 positive staining was observed in the MDM2 knockdown xenograft tumor, while lower expressions of PARP and cleaved caspase-8 were found in G5-DAT_62_/siScr treated tumor (Figure 5A). These results indicated that MDM2 knockdown induced cell apoptosis and inhibited the tumor growth. Moreover, MDM2 inhibition induced cell apoptosis was also supported by western blot analysis. As shown in Figure 5B, MDM2 knockdown significantly increased cleaved PARP and cleaved caspase-8 levels compared with the G5-DAT_62_/siScr group. Therefore, we assume that the inhibition of tumor growth in PC9-luc xenograft tumor was likely due to MDM2 knockdown induced apoptosis.

## DISCUSSION

RNA interference technology is new therapeutic strategy in the treatment of cancer due to its selectivity for targeted gene silencing. However, free siRNA are limited in RNA interference for its instability and poor bioavailability. In our study, we used a DAT-modified dendrimer G5-DAT62 that fulfills the two major requirements in siRNA delivery—high transfection efficacy and minimal cytotoxicity to knockdown the MDM2 gene.

Caspases are a family of proteins important for maintaining homeostasis through regulating cell apoptosis. Accordingly, caspases have been broadly classified by their known functions in apoptosis (caspase-3, -6, -7, -8, and -9 in mammals), and in inflammation (caspase-1, -4, -5, -12 in humans and caspase-1, -11, and -12 in mice) [32] Caspase-8 stimulates extrinsic apoptosis and active caspase-8 can initiate apoptosis directly by cleaving and thereby activating executioner caspase-3, caspase-6 and caspase-7 to induce efficient cell death [32].

Wild type p53 is a tumor suppressor and abrogating p53 function is a key event in human cancer, leading to activated cell cycle, genomic instability, resistance to stress signals, and ultimately leading to cancer development [34]. The p53 mutations occurs in half the cases of cancers and mutant p53 fails to emulate the transcriptional program to provide a robust response to stress. The oncoprotein MDM2 is a negative regulator of wild type p53 activity. MDM2 blocks the binding with wild type p53 with the transcriptional apparatus [35], mediates translocation of wild type p53 to the cytoplasm [36]. Furthermore, MDM2 can function as an E3 ligase to ubiquitinate p53 [37] and degrade wild type p53. In addition, MDM2 has been verified as a p53 inhibition gene, creating an autoregulatory feedback loop [38]. However, mutant p53 does not form a feedback loop with MDM2, as it is incapable of inducing MDM2 transcription. Therefore, only wild type p53 can be inhibited by the MDM2. It is reported that PC9 cells has mutated p53 version [39]. In our study, we showed that silencing MDM2 has a slight effect on p53 expression in PC9 cells. Therefore, there is a possibility that MDM2 repression induces apoptosis through a p53-independent pathway. MDM2 binds with other growth regulatory proteins including the Rb and ARF tumor suppressors which promote cell-cycle progression [28, 40, 41]. These binding likely contribute to inhibit the function of MDM2 in oncogenesis. The Rb is involved in cell cycle and differentiation by modulating the activity of transcription factors such as E2F protein family [42, 43]. In addition, MDM2 can induce NF-kB/p65 expression transcriptionally, through Sp1-binding sites, to induce resistance to apoptosis [44, 45]. Our finding that cell apoptosis is mainly caused by MDM2-Rb binding was confirmed in PC9 cells.

In summary, we found that G5-DAT_62_/siMDM2 polyplexes could effectively silence MDM2 expression in PC9 cells and induce cell apoptosis *in vitro*. The tumor growth was markedly inhibited in PC9 xenograft tumor model through inhibition of MDM2. These results suggested that the non-viral gene vector G5-DAT_62_ provide a therapeutic option to deliver siMDM2 for NSCLC gene therapy.

## MATERIALS AND METHODS

### Materials

Ethylenediamine-cored and amine-terminated G5 PAMAM dendrimer with a molecular weight of 28826 Da was purchased from Dendritech, Inc. (Midland, MI). 2-chloro-4,6-diamino-1,3,5-triazine was obtained from Aladdin Co. (Shanghai, China). Ethanol and sodium bicarbonate were purchased from Sinopharm Chemical Reagent Co., Ltd (Shanghai, China). Dimethyl sulfoxide-D6 (DMSO-D6) was obtained from Sigma-Aldrich (St. Louis, MO). siMDM2 (sense strand, 5′-GCUUCGGAACAAGAGACUC-3′) and scrambled siRNA non-specific to MDM2 (siScr, sense strand, 5′- GAUUAUGUCCGGUUAUGUAUU-3-3′) were purchased from GenePharma Co., Ltd (Shanghai, China). Lipofectamine 2000 (Lipo 2000) was obtained from Life Technologies (Shanghai, China). Antibodies were purchased from Santa Cruz (MDM2 and p53), Cell Signaling (cleaved PARP, cleaved caspase-8 and Rb) and Sigma (β-actin). G5PAMAM dendrimer was received in water and lyophilized to remove the solvent before further material synthesis. All the other chemicals were used as received without further purification.

### Synthesis of DAT-modified G5 PAMAM dendrimer

DAT-modified dendrimer was synthesized by a previously reported method [22]. Briefly, G5 PAMAM dendrimer and 2-chloro-4,6-diamino-1,3,5-triazine was dissolved in an ethanol-water solution (50%, v/v). The molar ratio of 2-chloro-4,6-diamino-1,3,5-triazine to G5 dendrimer is 77. Sodium bicarbonate was added to remove the yielding hydrochloric acid. The solution was stirred at 80°C for 24 h and intensively dialyzed against double-distilled water (molecular weight cut off, 3500 Da). Finally, the product was lyophilized as white powders and characterized by ^1^H NMR in DMSO-D6 (699.804 MHz, Bruker).

### Preparation and characterization of polyplexes

The synthesized materials were mixed with siRNA in diethylpyrocarbonate-treated water at different N/P ratios for 30 min. Nitrogen number (N) equals to the number of residual primary amine groups on each dendrimer, phosphorous number (P) equals to the number of phosphate groups in the DNA backbone. Since the two amine groups on DAT are not protonated at pH 7.4, they were not considered when calculating the N number. The size and zeta potential of the yielding complexes were measured by dynamic light scattering (DLS) (Zetasizer Nano ZS, Malvern, UK) and transmission electron microscope (TEM) (HT7700, HITACHI, Japan). siRNA binding ability of the DAT-modified G5 dendrimer was evaluated by an agarose gel retardation assay. The polyplexes prepared at different N/P ratios were diluted with DNA loading buffer and run on a 1.5% w/v agarose gel at 90 V for 15 min. The ethidium bromide stained gel was photographed using an UVIpro Gel documentation system (Tanon-2500, China).

### Cell culture

PC9 cells (a human lung cancer cell line) were purchased from ATCC. PC9 cells stably expressing a luciferase reporter gene (PC9-luc) were generated by transfecting pcDNA3.1-luc plasmid into the cells. The cells were grown in RP1640 medium supplemented with 100 units/mL penicillin sulphate, 100 μg/mL streptomycin and 10% heat-inactived fetal calf serum at 37°C in a humidified 5% CO_2_ and 95% air atmosphere.

### RNA interference and RNA analysis

PC9-luc cells were seeded at a density of 10^5^ cells per well in 12-well plates. The cells were cultured for 24 h before RNA interference experiments. Generally, dendrimer/siRNA polyplexes were prepared at different N/P ratios. The amount of siMDM2 in each well is almost fixed at 0.132 μg. The polyplex solution was diluted with 100 μL serum-free media and equilibrated at room temperature for 30 min. Then, the solution was further diluted with 100 μL serum-free media and incubated with the PC9 cells. After 6 h incubation, the cells were added with 500 μL medium (10% fetal calf serum). The RNA interference experiments were further continued for 42h. Parallel experiments were carried out using siScr that is inactive in silencing MDM2 as a negative control for the DAT-modified dendrimer. The commercial transfection reagent Lipo 2000 was tested as a positive control. Three repeats were conducted for each sample.

The transfected PC9-luc cells were homogenized in 1mL lysis buffer (TAKARA). The total RNA in the cells was extracted and 2μgof the RNA was reversely transcribed into cDNA with Moloney murine leukemia virus (M-MLV) reverse transcriptase (Invitrogen) following the manufacturer's instruction. The cDNA was subjected to real time-PCR analysis inhibiting MDM2 using SYBR Green PCR Mix (TOYOBO, Osaka, Japan). The MDM2-specific primers are as follows: MDM2 forward primer, 5′-GAATCATCGGACTCAGGTACATC-3′; MDM2 reverse primer, 5′-TCTGTCTCACTAATTGCTCTCCT-3′. The data were normalized to an endogenous reference (β-actin, forward primer, 5′-CGTCATACTCCTGCTTGCTG-3′; reverse primer, 5′-GTACGCCAACACAGTGCTG-3′.), and relative to that of untreated cells.

### Apoptosis detection in PC9-luc cells

Cell apoptosis was determined by Annexin V-FITC/PI Apoptosis Detection Kit (Abcam, Cambridge, MA, USA) according to the manufacturer's protocol. After transfection for 48 h, the cells were washed with cold PBS, re-suspended in 200 μL binding buffer, and incubated with 5 μL Annexin V-FITC and 5 μL PI solutions for 15 min at 4°C in the dark. After that, the cells were washed with cold PBS for three times and the cell apoptosis was analyzed by flow cytometry (FC500, Beckman Coulter, USA).

### Intracellular localization of G5-DAT_62_/siRNA polyplexes

PC9 cells were incubated with the FAM-labeled polyplexes for 1 h or 4 h. Acidic vesicles in the cells were stained with LysoTracker Red (DND-99, Invitrogen) and the cell nuclei were stained with Hoechst 33342. After incubation, the cells were washed with PBS for three times and the intracellular localizations of G5-DAT_62_/FAM-siRNA polyplexes were observed by a laser scanning confocal microscopy (Leica SP5, Germany).

### Cytotoxicity of G5-DAT_62_ on PC9 cells

The cytotoxicity of G5-DAT_62_ on PC9 cells was measured by a well-established 3-(4.5-dimethylthiazol-2-yl)-2, 5-diphenyltetrazolium bromide (MTT) assay. PC9 cells were incubated with G5-DAT_62_ at different concentrations (0-100 μg/mL) for 24 h. Five repeats were conducted for each sample.

### Hemolysis activity of G5-DAT_62_

Blood was drawn from BALB/c nude mice obtained from SLAC Laboratory Animal Co. Ltd. (Shanghai, China). The experiment was conducted according to the regulations of Association for Assessment and Accreditation of Laboratory Animal Care in Shanghai. The red blood cells were isolated form the blood by centrifugation at 2000 rpm for 5 min and washed with PBS for three times. Each test sample was incubated with red blood cells suspension (2%) diluted with PBS at 37°C for 1 h. PBS and Triton X-100 (0.5%) were used as negative and positive controls, respectively. The solutions were centrifuged for 2000 rpm for 5 min and the supernatant of all samples was measured at 545 nm to measure the absorbance of each sample.

### Animals and *in vivo* RNA interference

Female BALB/c nude mice (5 weeks) were purchased from SLAC Laboratory Animal Co. Ltd. (Shanghai, China). The animals were housed under specific-pathogen-free conditions within the experimental animal center at East China Normal University (ECNU). The animal experiments were carried out according to the NIH for care and use of laboratory animals and approved by the ethics committee of ECNU. PC9-luc cells were subcutaneously injected into the flank region of the mice. The mice were monitored by palpation for tumor induction. The animals were randomly divided into three groups with five mice in each group when tumor size reaches around 200 mm^3^. The tumor-bearing mice were administrated with PBS, dendrimer/siRNA-MDM2 and dendrimer/siRNA-Scr polyplexes (containing 2 μg siMDM2 or siScr, the polyplexes were prepared at the optimal N/P ratio of 100) by injection into the tumor, and the treatments were repeated every other day until the fourteen day. The tumor volume and body weight of the mice were recorded until the fourteen day. For *in vivo* luminescence imaging, the tumor-bearing mice were anesthetized with isoflurane and intraperitoneally injected with D-luciferin at a dose of 200 mg/kg. After 5 min, the luminescence at tumor site was recorded using an IVIS imaging system (Xenogen, Alameda, CA). After luminescence imaging, all the mice were euthanized, and the tumors were isolated and the weights of the tumors were measured.

### Western blot analysis

Transfected PC9-luc cells were washed with cold PBS for three times and homogenized in a lysis buffer. The cell lysates were separated on 10% SDS-PAGE gels and transferred onto a PVDF membrane. The membrane was incubated with monoclonal antibodies against MDM2 and cleaved caspase-8 overnight at 4°C, and further incubated with fluorescently labeled secondary antibody for 1 h. The protein bands were visualized using the Odyssey Infrared Imaging System (LI-COR, USA). β-actin was selected as the endogenous reference.

Tumor tissues were homogenized in liquid nitrogen and lysed for 30min in ice-cold protein extraction buffer. Equivalent amount of total protein from each sample was loaded and immune-blots were analyzed using primary antibodies specific forMDM2, PARP, p53, Rb and cleaved caspase-8, p53and Rb overnight. After incubation with fluorescent labeled secondary antibody, specific protein bands were visualized by the Odyssey Infrared Imaging System.

### Immunohistochemistry (IHC)

Tumor tissues were fixed with 4% paraformaldehyde for 3 days and then dehydrated through a graded series of ethanol, embedded in paraffin and sectioned at 4μm. The sections were dewaxed, rehydrated, and stained with hematoxylin and eosin. IHC was performed following standard histological procedures described in the manual for Histostain-Plus (DAB) IHC kit (Mingrui Biotech, Shanghai).

### Data collection and statistical analysis

The statistical data was obtained by GraphPad Prism v5.0 software. Statistical analysis was performed using two tailed, paired Student's t-test. A p-value of less than 0.05 was considered statistically significant.
